# Engaging with diverse audiences to raise awareness about childhood eczema: reflections from two community events

**DOI:** 10.1186/s40900-021-00251-8

**Published:** 2021-01-19

**Authors:** Anna Gilbertson, Matthew J. Ridd, Eileen Sutton, Lyn Liddiard, Julie Clayton, Amanda Roberts, Jonathan Chan, Alisha Bhanot, Rosie Wellesley, Shoba Dawson

**Affiliations:** 1 Centre for Academic Primary Care, Population Health Sciences, Bristol Medical School, University of Bristol, Bristol, UK; 2 Centre of Evidence Based Dermatology, Division of Rheumatology, Orthopaedics and Dermatology, School of Medicine, University of Nottingham, Nottingham, UK; 3 General Practitioner and Illustrator, Herefordshire CCG, London, UK

**Keywords:** Eczema, PPI, Diversity, Public engagement, Creative approaches

## Abstract

**Background:**

Eczema is a common childhood condition, causing dry and itchy skin which can be difficult to manage. We have been undertaking eczema and food allergy research to address previously prioritised research questions. We obtained funding to trial novel approaches to reach diverse audiences to raise awareness of childhood eczema, research, and public involvement in research.

**Methods:**

This paper reflects on two public engagement events held in collaboration with stakeholders in two settings of ethnic diversity in East Bristol, UK. We invited parents and children to attend the events by public display of posters. We created novel activities related to the research and involved artists to engage parents/carers and children about eczema and the research we are doing into its management.

**Results:**

Attendance at the first event was lower than expected. Lessons learned were incorporated into the second event, to use a more structured approach and attract greater numbers of parents/carers from more diverse backgrounds. Creative approaches such as using artists at both events made the subject more accessible for diverse audiences, including children.

**Conclusion:**

We successfully delivered two public engagement events. The success of the events has generated individual interest in PPI and enquiries about future events from neighbouring community groups. Reflections from the events have also been fed back to inform the research.

## Plain English summary

Eczema is a common childhood condition, which can be difficult to manage. We are leading two research studies to answer uncertainties about eczema treatment. Firstly, the Best Emollients for Eczema (BEE) study, is comparing how well different types of emollients work. The main treatment for the dry skin is moisturisers (or emollients as they are known medically) but there are many different types, and a common question is if one type is better than another. Secondly, the Trial of Eczema allergy Screening Tests (TEST), is looking at whether food allergy test-guided dietary advice improves eczema. Parents often ask if a food allergy is causing their child’s eczema and although food allergy is more common in children with eczema, there is disagreement over whether it causes long-term symptoms. We work closely with Patient and Public Involvement (PPI) contributors to help us set-up and run these studies. To share our work with people who we might not otherwise reach, we organised two events in community settings in East Bristol, UK and invited parents and children, with and without eczema, to attend. Those who attended, engaged with the activities and the research team. Following the success of the events, parents/carers from diverse backgrounds have expressed interest in contributing to future eczema research in PPI roles, and neighbouring community settings have requested another eczema awareness event. In addition, reflections from the events have been fed back into the research.

## Background

Atopic eczema/dermatitis (hereafter eczema) is a common itchy skin condition affecting around 20% of children [1]. In the UK, childhood eczema is mostly diagnosed and managed in primary care [2] but symptoms can be troublesome and have a significant impact on family quality of life [3]. The dermatology primary care research team at University of Bristol have led on two studies related to childhood eczema management: the research questions behind both studies arose from the James Lind Alliance eczema research priority setting partnership (2013), which facilitates collaboration between clinicians and patients to identify uncertainties and prioritise future research [4].

The Best Emollient for Eczema (BEE) study is a National Institute for Health Research (NIHR) Health Technology Assessment (HTA) funded multi-centre, individually randomised controlled trial comparing the effectiveness and acceptability of four different types of emollients commonly used to treat eczema in children. More about this study is detailed elsewhere [5] but in brief, 550 children aged 6 months to 12 years were recruited via GP surgeries between January 2018 and October 2019. Participants were randomised to use either a lotion, cream, gel or ointment as their only leave-on emollient. Parents/carers (hereafter parents) were asked to assess their child’s skin, using the Patient Orientated Eczema Measure (POEM), weekly over the 16-week primary outcome period and monthly thereafter for the total 52-week follow-up period. A nested qualitative study, comprising interviews with parents and older children, will complement and aid the understanding of the quantitative findings regarding the perceived benefits or problems with the study emollients.

The TEST (Trial of Eczema allergy Screening Tests) study, funded by the NIHR School for Primary Care Research (SPCR), was a single-centre randomised controlled trial with nested qualitative study that proposed to determine the feasibility of a trial comparing test-guided dietary advice to current standard practice. Again, more information about this study is detailed elsewhere [6]. In brief, 84 children age 3 months – 5 years were recruited from GP surgeries between September 2018 and February 2019 and participants were randomised to intervention (structured allergy history and skin prick allergy test) or usual care. Participants were followed up for six months post baseline, by means of a monthly diary and follow-up visit. The nested qualitative study included interviews to help provide a better understanding of issues of food allergy, allergy tests and dietary modifications in children with eczema. An application for a full clinical trial is in progress, following the success of the feasibility trial.

### Patient and public involvement and engagement (PPIE)

PPI entails research carried out ‘with’ or ‘by’ members of the public rather than ‘to’, ‘for’ or ‘about’ them [7]. Public engagement is where information and knowledge about research is provided and disseminated [7]. However, involvement and engagement can be viewed as a part of a continuum, as engagement can be the first step to initiating involvement and related activities. Another example would be to disseminate findings with the public where engagement can be the endpoint of involvement.

PPI has been an integral part of both BEE and TEST. For the BEE study, we worked with a group of contributors with one contributing to discussions at Trial Management Group (TMG) meetings and one contributing to Trial Steering Committee (TSC) meetings. The study has also received PPI support via the Patient Panel of the UK Dermatology Clinical Trials Network’s Centre of Evidence Based Dermatology. All contributors are parents of children with eczema. PPI involvement included testing the parent-facing questionnaires for logic and clarity and providing feedback on the children’s participant information sheet and lay summary.

In the TEST study, the same local group of contributors were involved throughout the study process; for example, two mothers of children with eczema and food allergy contributed at TMG meetings, contributors were asked to review paperwork, and participate in baseline visit rehearsals. One contributor sat on the TSC and contributed regularly to discussions. Both studies share their research activity via regularly updated Twitter feeds and PPI contributors have been involved in the design and content of study newsletters.

### The NIHR SPCR patient and public engagement project

This project was funded by the 2019 NIHR SPCR Patient and Public Engagement (PPIE) grant. The award was intended to encourage researchers within the SPCR Schools to trial novel approaches to PPI and/or public engagement or extend their work to reach new audiences.

#### Aim

The aim of this project was to raise awareness of eczema, the challenges of treating it, and the current research addressing uncertainties in eczema management. We sought to achieve this by undertaking two public engagement events, which would also be an opportunity to invite individuals to contribute to future research planning and dissemination. This paper provides a descriptive account of the public engagement events undertaken by the research team including our reflections and the lessons learned.

#### Objectives

Our objectives were:

- To use creative, co-produced approaches to raise awareness and engage with children with eczema and their parents.
- To reach diverse audiences through undertaking public engagement events and promote inclusive PPI and participation and/or contribution in future research.

## Methods

The research team included an Academic GP (MR- Chief Investigator of the BEE and TEST studies), researchers (ES, SD – both with experience of leading PPI), research nurses who worked on the BEE and TEST studies (LL, AG), a PPI contributor to the BEE study (AR), a GP and children’s author (RW) medical students (JoC, AB) and a PPI coordinator (JC). Led by SD, we planned and conducted two public engagement events in collaboration with community centre organisers in East Bristol, which is an area densely populated with Black, Asian and Minority Ethnic (BAME) families [8].

The first event was part of ‘Fun Palaces’. Fun Palaces is an initiative designed to support people to co-create cultural and community events across the UK and worldwide and is run by and for local communities (https://funpalaces.co.uk/about-fun-palaces/). The Public Engagement Team of the Elizabeth Blackwell Institute, University of Bristol, was responsible for hosting the local Fun Palace event which took place at Barton Hill Settlement, Bristol on Saturday 5th October 2019. We set up an ‘Eczema Awareness Fun Palace’ and advertised this event through different platforms such as Eczema Outreach Support newsletter, upyourstreet Bristol (https://upourstreet.org.uk/), local PPIE coordinators, University of Bristol weekly newsletter and Twitter. Ten families attended this event over the course of the six-hour (10 am-4 pm) day.

The second event was undertaken in a local community group setting at St. Werburgh’s Community Centre, East Bristol. The community group was set up to support BAME families with children with disabilities. We worked with the community group organisers to understand what sort of activities should be undertaken to effectively engage with the audience. With it being a weekday, the children’s craft activities were not thought to be needed, and the role-play was not recommended in case of translation difficulties. They also advised where to display posters to advertise the event, which included local libraries, community groups, learning centre, local schools, children’s gymnasium, GP surgeries, pharmacies, and health centres. Twenty-four families attended the two-hour event (12-2 pm) on Monday 20th January 2020.

While the first event (Fun Palaces) was held in multiple locations across Bristol, we chose the Barton Hill Settlement, and the second event was held at the St Werburgh’s Community Centre because these areas have a high proportion of ethnic minority population and also form part of a socially and economically disadvantaged area. These areas were also chosen as there is a lack of inclusion of people from these groups in PPIE activities. For both the events, efforts were made to reach diverse audiences; therefore, we advertised and promoted these events through various platforms as described above. The events were free and open to all to attend. In preparation for the events, we collectively devised creative approaches based on our experiences of interacting with participants as research nurses (AG, LL), clinical scenarios between GP (MR) and eczema patients, and parent/carer experiences (AR). For example, AR helped design and led a card-based activity to engage and encourage children to talk about how different seasons and materials can affect eczema. To promote and optimise engagement we collaborated with artists and national organisations (e.g., Eczema Outreach Support) by promoting the event and accessing approved information sheets about eczema to share at the event. During this project, we held 10 meetings to plan and develop activities for the respective events as well as to reflect on what did and did not go well and what could have been done differently after each event. We had a final reflective meeting at the end of the project to discuss lessons learned. We did not formally evaluate impact of PPIE as this approach often fails to describe how patients and public have influenced research and certain types of impacts such as empowering patients etc. are not always captured through these means [9]. Rather, this process was about mutual learning between researchers and PPIE contributors; therefore we adopted a reflective approach to highlight not only the positive but also any potential negative learning experiences from different perspectives.

## Results

First, we provide a descriptive account of the processes involved in designing and undertaking these events. This is followed by the team’s reflections and lessons learned.

### Event 1 - Fun Palaces

**Fig. 1:**
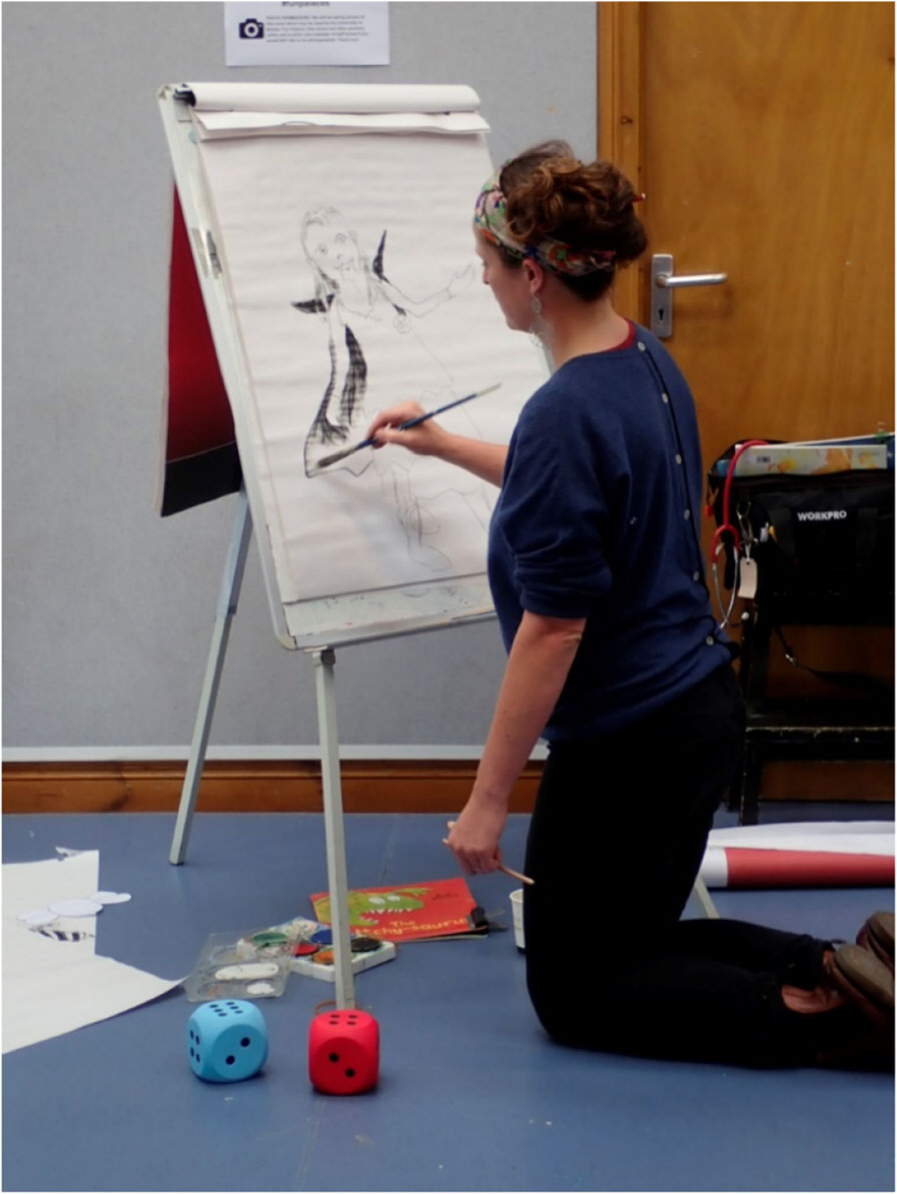
Children’s author and illustrator

The 10 families who attended this event were primarily White (White British/White European). Engagement activities at this event included a storytelling and illustration session run by co-author (RW), who is an artist and GP with an interest in eczema. Children were given free copies of the book ‘Itchysaurus’, authored and illustrated by Rosie to take home as well as a personalised illustration (Fig. 1).

**Fig. 2:**
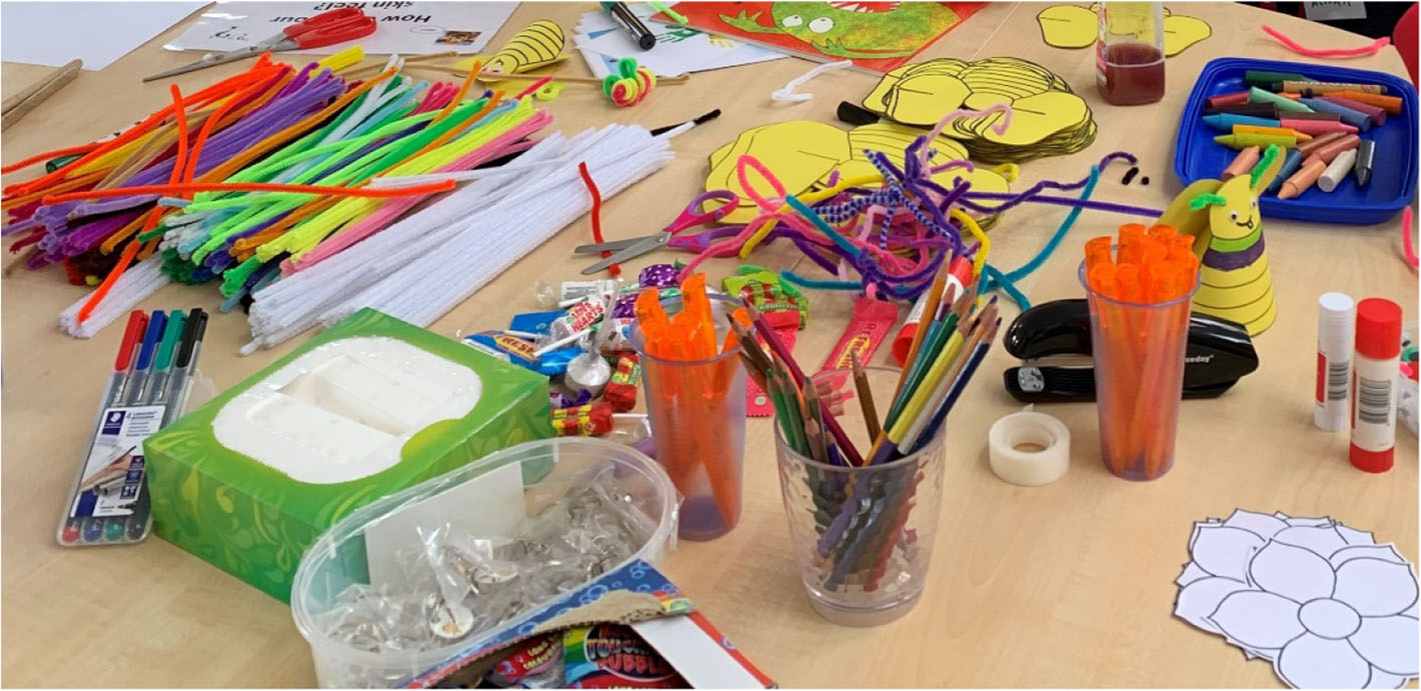
Children’s craft activities

Parents and children also had the opportunity to informally chat with the research team and find out about our current research. In addition, resources from Eczema Outreach Support were available on topics such as eczema management. AR was at this event to share her experiences of living with eczema, being a carer of children with eczema and being involved in research as a PPI contributor. Children were invited to participate in different arts and crafts-based activities linked to the research and appropriate for the age of the children. This included an activity based on the BEE Study, which involved children colouring in a bee and sticking it on the cream, gel, ointment or lotion ‘hive’, which they thought was ‘best’ for eczema. We also prepared materials for the children to create 3D ‘bees’ out of card and pipe cleaners (Fig. 2).

**Fig. 3:**
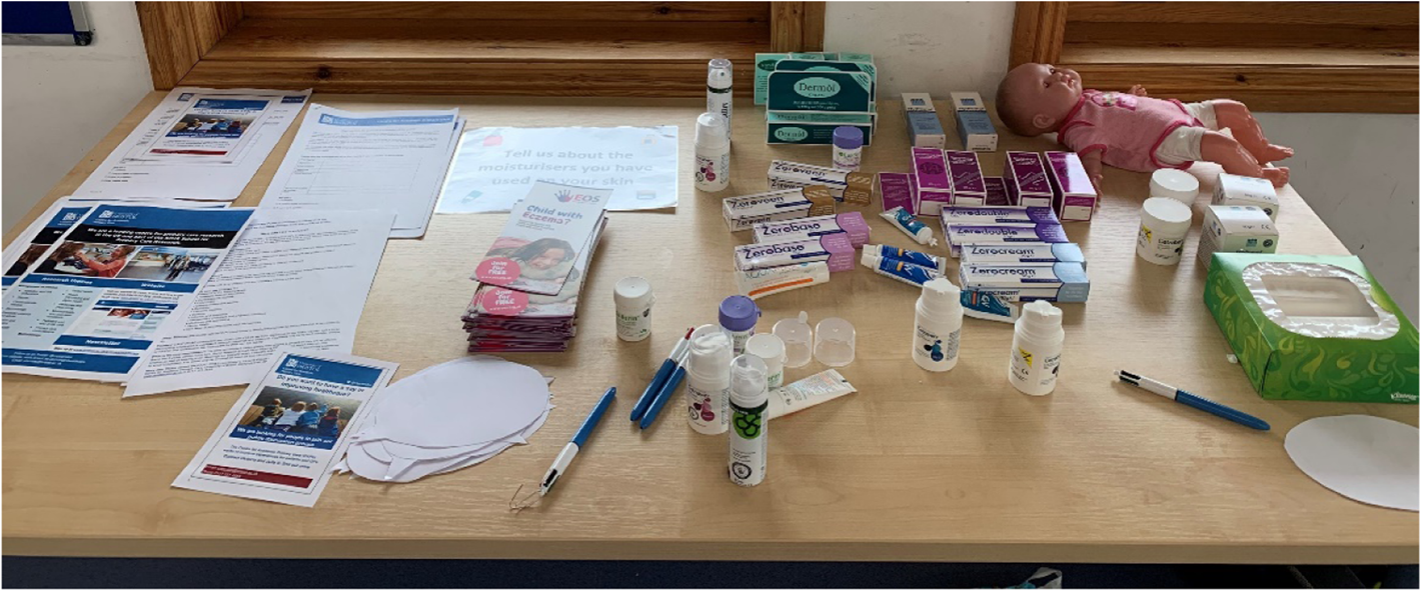
Emollient testing table

The children were also invited to participate in a card-based activity set up by AR to talk about how different weather and different materials that they are exposed to on a regular basis can affect eczema. Other activities included opportunities for children and carers to trial different types of emollients on dolls, facilitated by medical students (JC and AB), so that they could discuss their preferred emollient with us and get a sense of the different features of the different types (see Fig. 3).

Based on our clinical and research experiences, we felt that those attending might not be aware of the different types of emollients available or how to use them safely and effectively. At both events, participants had the opportunity to trial the different types of emollients and the technique on how to apply them safely and effectively was demonstrated. We also displayed images on the walls offering tips for effective emollient use.

We displayed study related posters and explained about PPI and related opportunities, therefore raising awareness about other ways to contribute to research. Large dice were used to explain the process of randomisation in clinical trials to children and carers who attended the event. A role play of common scenarios between patient and GP was prepared but the lack of structure and inconsistent flow of individuals attending, who did not all have eczema, meant this activity was not taken up.

#### Reflections with TEST and BEE study PPI contributors

Following the first event, we shared our experiences and lessons learned with the BEE and TEST PPI contributors and asked for their suggestions on how to improve the second event. Their suggestions included reducing the number of activities and adopting a more structured approach.

#### Event 2 - community engagement event

**Fig. 4:**
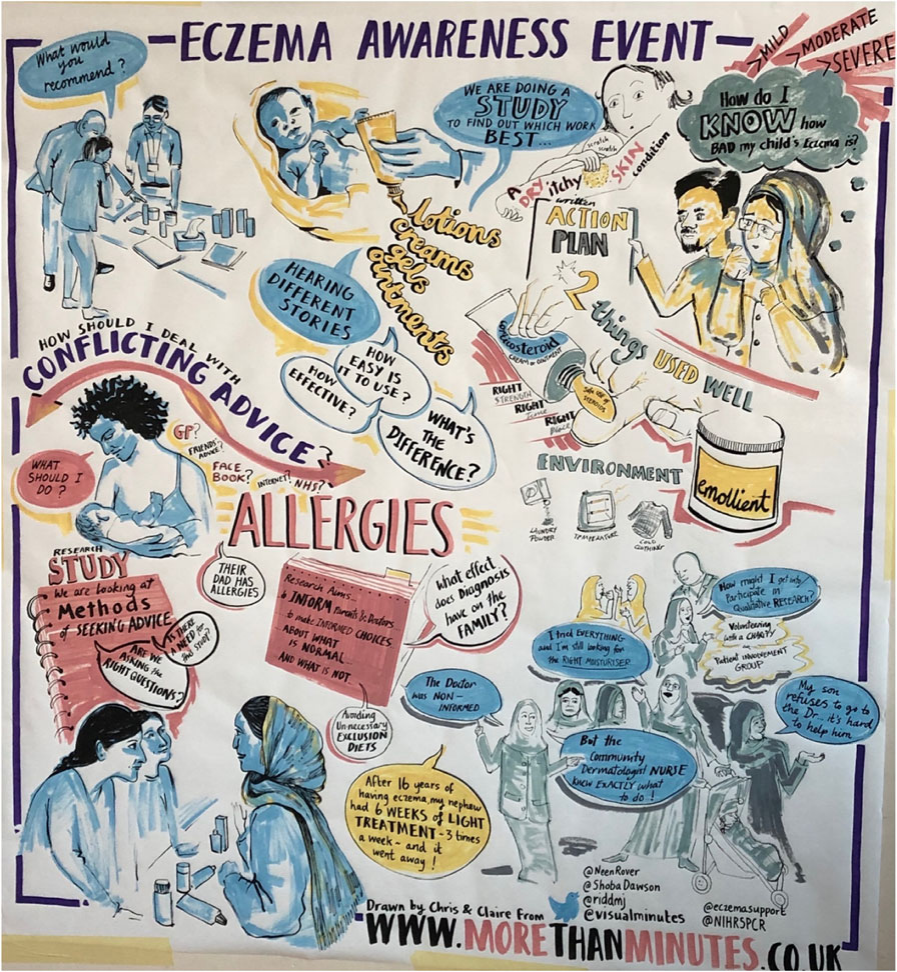
Visual minutes of discussions from the community engagement event

Those who attended the event were primarily of South Asian origin. Community group organisers acted as interpreters throughout the event where needed. This event was more structured, starting with three members of the research team (MR, ES & AG) each delivering short PowerPoint presentations about eczema, the importance of research like the BEE and TEST studies, and the importance of seeking opinions of parents and children in future research. The audience were invited to ask questions at any point, which they did. The presentations were followed by opportunities for the families to meet with the research team and discuss the condition, test different emollients and learn more about research participation and involvement opportunities. Artists from Visual Minutes were present to record the discussions during the event (Fig. 4). This was electronically shared with the community group and families that attended the event.

#### Reflections

We met regularly in preparation for the events but also afterwards to reflect on what went well and what did not. Themes from these reflective meetings and individual quotes are detailed below.

*“Being part of this outreach team has been a privilege and joy. I was involved during the preparation of the funding bid and continued to be included in all the planning for the two events both by email, telephone conferences and one-to-one conversations with Shoba (SD). My suggestions were welcomed and acted upon. I was only able to attend the first, early October 19 Fun Palaces event.” (AR, PPI Contributor)*

#### Fun palaces event

On the Saturday that the Fun Palace Event was held, there was little passing footfall other than to/from an event in an adjoining room, from which the majority of families were drawn. Although they were mostly white and well-educated, their experience of eczema and food allergy differed, with some having no experience at all previously. We welcomed families into and through the door, talking and guiding them through the range of different activities. The craft activities were popular with children and freed parents to talk to researchers about eczema and/or research if they wished.

For the older children who could understand why the event was occurring, we felt that they found the event fun and appreciated that research was being done to try to help them. Working together on the crafts the children were able to talk about how life was for them with their eczema in a fun, non-threatening environment.

*“I was struck by the honesty of the audiences in talking about the difficulties in managing their eczema and the effect this has on their lives. I saw first-hand and gained an appreciation for how PPI shapes future research in identifying patient priorities.” (AB, Medical Student)*

However, overall, this event did not go as well as hoped. The room was spacious and light but physically distant from the rest of the community centre and it was not easy to display materials on the walls as anticipated. The activities and materials had been designed to engage with local families, but attendance was lower than expected and the research team out-numbered the number of visitors throughout the day. Interestingly, half of the families we spoke to had not experienced eczema themselves but were still willing to engage in the activities and with the team.

*“At the first event I was there to share my experience of being involved in research and of eczema as a carer (I help run a support group for carers of children with eczema, too). But I also delighted in hearing the carer’s journeys as I talked to them on an individual basis.” (AR, PPI Contributor)*

On reflection, we recognised that we could not rely on ‘piggy backing’ onto promotion of the Fun Palaces to ensure a good attendance at our own event; that the physical location of the venue was important; and that a ‘more is less’ approach may be appropriate in terms of what’s offered to attendees.

#### Community engagement event

The second event was better attended because there was a ‘captive audience’ of families attending a regular group and additional families who had seen flyers of the event.

*“I was surprised and delighted that, what I viewed a more traditional approach of ‘talk and chalk’ (Power Point!), was wanted and received so well, and that we had such a large, ethnically diverse audience who wanted to engage with questions about the research and what this meant for them and their families. It was also much more ‘efficient’ for the team than the first event, partly because we’d developed the materials for the first event, but also because we had a set start time for the talks with time-bound room for discussion afterwards.” (MR, Chief Investigator)*

The audience engaged with the presentations and asked questions about our work, particularly as there were more families whose children had eczema. After the interactive presentations, parents talked to the research team individually, either about their experiences of trying samples of different emollients displayed or about the research. The children’s activities were not needed at this event as it was predominantly attended by parents, although in hindsight some young baby toys would have been helpful.

Many parents were looking for our advice on which emollient was best, which opened an opportunity to explain further why the BEE research on this topic is needed. At both events parents were inquisitive about the name of the BEE study, expecting there to be a ‘bee’ or honey component to treating eczema, which has been taken on board. We also noticed how people mainly call emollients ‘creams’ despite there being the different types, which is consistent to what we have observed in our research. It was also notable how parents/children quickly recognised some brands by name or packaging but did not necessarily distinguish or appreciate that some were available in different types, e.g. Epaderm cream and Epaderm ointment (a variety of brands, as well as types, were available to try). All these topics were good starting points for individual conversations about the research and to reinforced findings from the research itself.

*“My reflections from the day (apart from how hard everyone worked to make the day successful) helped with changes for the second event and also helped us all to understand the inadvertent result of choosing BEE (Best Eczema Emollients) as the acronym for one of the studies for which this project built awareness. It raised the question in people's minds as to whether bees and eczema were therapeutically connected.” (AR, PPI Contributor)*

Through running the two events in these areas of Bristol, we aimed to reach diverse audiences. While efforts were made to maximise the participation and engagement of people from diverse backgrounds, we were not able to achieve this for the first event. We were able to target and reach certain groups who are seldom engaged in PPIE activities for the second event.

The doctor-patient role play was not recommended by the community group organisers for the second event due to perceptions that it would be less effective in engaging with the audience and potential translation issues. An alternative for future consideration would be to have a pre-recorded version of a doctor-patient consultation role-play which could then be translated by the interpreter on the day or for which a written translation can be provided.

We have received feedback from the community group organiser that the second event was informative and effective at enhancing the group’s knowledge of managing eczema. The Visual Minutes taken of the meeting discussions at the event were also said to be effective at reducing barriers to engagement and enhancing discussions. Parents who attended have told us they have since changed their child’s moisturiser to a different type. Although the majority of those who attended the second event were part of the existing group, a significant number attended having seen the advertisements elsewhere and expressed an interest in contributing to future research.

*“As a Patient and Public Involvement (PPI) and Engagement coordinator for the Centre for Academic Primary Care, these events represented an opportunity to make contact with community representatives that we had not engaged with before, and venues that were new to us, and we shall continue to offer opportunities for future public involvement and engagement through these same networks. It was gratifying to meet new members of the Central and East Bristol communities who attended the two events, and to discuss the potential for their involvement in future research. Six of the parents expressed interest in joining our CAPC ‘pool’ of public contributors, and one has subsequently been involved in a PPI meeting for the TEST study.” (JC, PPI Coordinator)*

The events were also a positive experience for the research team, many of whom had not been involved in PPI and engagement at this level before.

*“It was a privilege to be involved in the public engagement events. I have gained valuable skills in communication and have a greater understanding of the difficulties in managing a chronic condition and the fundamental importance of PPI, all of which will help me to become a better academic clinician.” (AB, Medical Student)*

#### Lessons learned

- Working collaboratively with PPI contributors and existing community groups ensures the event is applicable to the needs of an established audience.
- Advertising in local community settings such as schools and libraries as well as social media is effective at broadening the reach of awareness events.
- A structured approach works well by providing opportunities to address an audience and talk to individuals within specified time frames.
- Using creative approaches such as artists make topics more accessible for diverse audiences. These informal approaches reduce barriers to engagement and enhance discussions about the research topics.
- Asking attendees to complete an evaluation form would provide quantifiable evidence of what they did and didn’t find valuable at the events, which may help ensure success of future events.
- Establishing interest or issuing tickets for events may be useful to determine the number of people expected to attend and plan resources accordingly.

## Conclusion

We delivered two public engagement events, using novel approaches to raise awareness of childhood eczema management and public involvement in research. By reflecting on the limited reach of the first event, the second event was adapted by using a structured approach and using our insight of the targeted group, to reach a diverse audience who had experience of the condition. There were components of both events that worked well, such as the involvement of artists to reduce barriers of engagement. Following the success of the events, parents have expressed interest in contributing to research in PPI roles. We have also received interest from a group of four Children’s Centres in East Bristol, who have requested an eczema awareness event for parents in their community. We have been successful in obtaining additional funding to achieve this and we are applying the lessons learned from these events, such as using a structured approach, establishing interest prior to events and working closely with the children’s centre organiser, to ensure the event is tailored to meet the needs of the audience. It was apparent from both these engagement events that the research we are doing and planning to do in the future is relevant to concerns raised by parents of children with eczema.

## Data Availability

Not applicable.

## Abbreviations

PPI: Patient and Public Involvement
PPIE: Patient and Public Involvement and Engagement
CAPC: Centre for Academic Primary Care
NIHR: National Institute for Health Research
TEST: Trial of Eczema allergy Screening Tests
BEE: Best Emollients for Eczema
GP: General Practitioner
UK: United Kingdom
BAME: Black, Asian and Minority Ethnic
TMG: Trial Management Group
TSC: Trial Steering Committee
HTA: Health Technology Assessment
POEM: Patient Orientated Eczema Measure
SPCR: School for Primary Care Research
